# Identification of an Amino Acid Metabolism-Related Gene Signature for Predicting Prognosis in Lung Adenocarcinoma

**DOI:** 10.3390/genes13122295

**Published:** 2022-12-06

**Authors:** Wuguang Chang, Hongmu Li, Chun Wu, Leqi Zhong, Tengfei Zhu, Zenghao Chang, Wei Ou, Siyu Wang

**Affiliations:** Department of Thoracic Surgery, Sun Yat-sen University Cancer Center, State Key Laboratory of Oncology in South China, Guangzhou 510060, China

**Keywords:** amino acid metabolism, prognosis, lung adenocarcinoma, immunotherapy, tumor microenvironment

## Abstract

Dysregulation of amino acid metabolism (AAM) is an important factor in cancer progression. This study intended to study the prognostic value of AAM-related genes in lung adenocarcinoma (LUAD). **Methods:** The mRNA expression profiles of LUAD datasets from The Cancer Genome Atlas (TCGA) and Gene Expression Omnibus (GEO) were applied as the training and validation sets. After identifying the differentially expressed AAM-related genes, an AAM-related gene signature (AAMRGS) was constructed and validated. Additionally, we systematically analyzed the differences in immune cell infiltration, biological pathways, immunotherapy response, and drug sensitivity between the two AAMRGS subgroups. **Results:** The prognosis-related signature was constructed on the grounds of key AAM-related genes. LUAD patients were divided into AAMRGS-high and -low groups. Patients in the two subgroups differed in prognosis, tumor microenvironment (TME), biological pathways, and sensitivity to chemotherapy and immunotherapy. The area under the receiver operating characteristics (ROC) and calibration curves showed good predictive ability for the nomogram. Analysis of immune cell infiltration revealed that the TME of the AAMRGS-low group was in a state of immune activation. **Conclusion:** We constructed an AAMRGS that could effectively predict prognosis and guide treatment strategies for patients with LUAD.

## 1. Introduction

LUAD is the most prevalent type of lung cancer, making up 40% of all cases of lung cancer [1]. Its 5-year survival rate is only 15% [2]. The early stage of LUAD can be detected by computed tomography (CT) [3] and then cured by radical surgical resection. However, in more than 50% of patients, LUAD has already metastasized upon initial diagnosis [4]. In the past decades, with further study of molecular targets, such as EGFR, ALK, and PD-1/PD-L1, targeted therapy and immunotherapy for LUAD have achieved remarkable results [5]. However, due to the heterogeneity of LUAD, only a tiny portion of patients benefit from such therapy [6]. These patients initially manage their disease with targeted therapy or immunotherapy; however, eventually, resistance inevitably develops [7]. The existing TNM staging does not reflect the molecular features of LUAD [8]. Therefore, it is necessary to identify new therapeutic targets through a large number of data analyses to provide individualized treatment plans for patients with LUAD.

The core characteristic of cancer is metabolic reprogramming [9]. Compared with normal cells, cancer cells require large amounts of energy for proliferation, invasion, and metastasis. The metabolism of amino acids and their derivatives is one of the key factors of cancer progression [10]. In addition, the function of immune cells in TME is affected by amino acid metabolism, leading to tumor immune escape [11]. As shown in previous studies, glutamine is an important nutrient for cancer cells. It not only participates in the energy metabolism of cancer cells but also protects cells from oxidative stress as an antioxidant [12]. Rober et al. found that blocking the metabolism of glutamine could effectively inhibit the metabolic activity of cancer cells, but the activity of effector T cells was not affected, so targeted glutamine therapy may become an effective measure to improve the efficacy of immunotherapy [13]. Liu et al. found that glutamine metabolism significantly affected the TME status and immunotherapy efficacy of LUAD using bioinformatics [14]. Branched-chain amino acids (valine, leucine, and isoleucine; BCAAs) are essential amino acids that promote the expression of mitochondria-related genes to enhance tumor proliferation and activate the mTOR signaling pathway to stimulate tumor growth [15]. Kayo et al. showed that the immunosuppressive effect of Treg cells in mice decreased after reducing the intake of BCAAs in mice [16]. These findings imply that tumor development is significantly influenced by amino acid metabolism. Thus, it has emerged as a potential target for cancer therapy. However, the potential value of amino acid metabolism-related genes in LUAD has not been analyzed.

To better understand the prognosis of LUAD patients and give them more individualized care, we developed an AAMRGS to evaluate immune cell infiltration and the response to immunotherapy and chemotherapy in LUAD patients.

## 2. Materials and Methods

### 2.1. Data Collection

After excluding patients with missing survival information, mRNA (TPM) data and corresponding clinical information of 496 LUAD and 59 normal samples were obtained from the TCGA. The TPM format was then converted to log2(TPM+1) for subsequent analysis. GSE72094 and GSE31210 from the GEO were collected as validation sets. AAM-related genes were obtained from the molecular signature database [17] (Table S1).

### 2.2. Differentially Expressed AAM-Related Genes and Enrichment Analysis

The “limma” R package (version 3.50.3) was employed for differential analysis of the LUAD and normal tissues from the TCGA [18]. The threshold was set to a false discovery rate (FDR) < 0.05 and log2 |fold change| ≥ 1. The “clusterProfiler” R package (version 4.2.2) was then used to implement GO and KEGG enrichment analysis of differentially expressed AAM-related genes [19].

### 2.3. Construction and Validation of the AAMRGS

To identify prognosis-related genes (*p* < 0.05), a univariate Cox regression analysis of the differentially expressed AAM-related genes was carried out. Subsequently, LASSO and multivariate Cox regression analyses were employed to identify the final key genes and their corresponding coefficients to set the following calculation: risk score = Coef1 × Gene1_exp_ + Coef2 × Gene2_exp_ + ⋯ Coefn × Genen_exp_. The TCGA and GEO cohorts were grouped according to low-risk (35%) and high-risk (65%). Kaplan–Meier (KM) survival analysis and log-rank tests were then performed. Time-dependent ROC curves were used to assess the predictive power of the signature.

### 2.4. Development of a Nomogram

To assess whether the AAMRGS was a reliable prognostic marker for LUAD patients, univariate and multivariate Cox regression analyses were performed. On the grounds of the results of the multivariate Cox regression using the “rms” R package (version 6.3-0), a nomogram was established. The predictive power of the nomogram was assessed by calibration curves and time-dependent ROC curves.

### 2.5. Gene Set Enrichment Analysis

To understand the differences in biological function and pathways between the AAMRGS-high and AAMRGS-low groups, GO and KEGG enrichment analyses were performed using GSEA (version: 4.2.3; Broad Institute, Inc., USA) [17]. The threshold was set at *p* < 0.05.

### 2.6. Immune Cell Infiltration Analysis

Single sample gene set enrichment analysis (ssGSEA) was employed with the “GSVA” R package (version 1.42.0) to analyze the degree of infiltration of 28 immune cells [20].

### 2.7. Prediction of Immunotherapy Response

The immunophenoscore (IPS) of LUAD cases was acquired from The Cancer Immunome Atlas (TCIA) database [21], which is an effective indicator for predicting the sensitivity of LUAD patients to anti-PD-1 and CTLA-4 immunotherapy. Furthermore, we used one immunotherapy dataset to evaluate the prediction ability of the signature: 298 patients with locally advanced or metastatic urothelial carcinoma who received anti-PD-L1 (atezolizumab) immunotherapy (IMvigor210) [22]. In the same way, the IMvigor210 cohort was grouped according to low risk (35%) and high risk (65%).

### 2.8. Drug Sensitivity Analysis

To analyze the sensitivity of the two AAMRGS subgroups to chemotherapeutic drugs, the “pRRophetic” R package (version 0.5) was utilized to calculate the half-maximal inhibitory concentration (IC50) of popular chemotherapeutic drugs [23].

### 2.9. Statistical Analysis

All statistical analyses were performed using SPSS (version 26.0; IBM, Chicago, IL, USA) and R software (version: 4.1.3; The University of Auckland, Auckland, New Zealand). Differences between the two AAMRGS subgroups were calculated with the Wilcoxon rank-sum test. Cox regression analysis was used to identify risk factors for prognosis in patients with LUAD. Spearman rank correlation was used for correlation analysis. The threshold of all statistical analyses was *p* < 0.05.

## 3. Results

### 3.1. Identification and Enrichment Analysis of Differentially Expressed AAM-Related Genes

**Figure 1:**
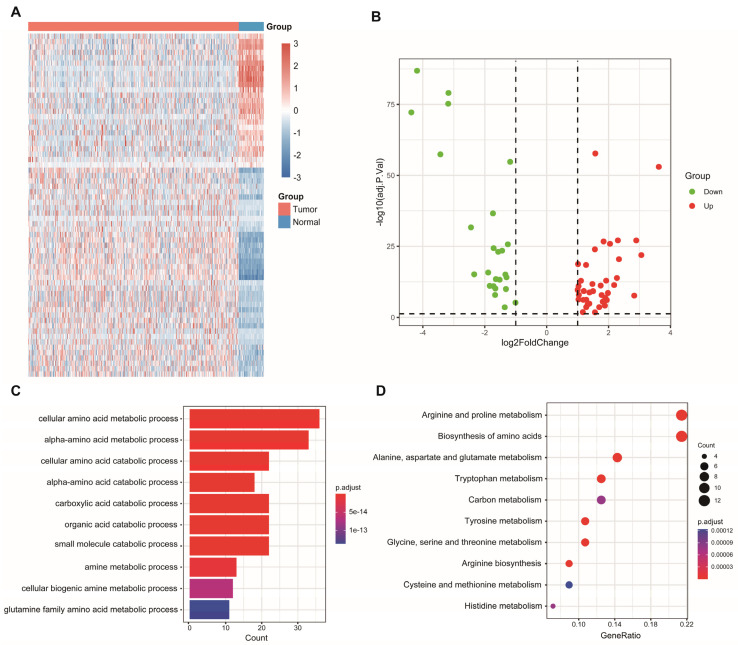
Identification and enrichment analysis of AAM-related genes in LUAD. (**A**) Heatmap showing 64 differentially expressed AAM-related genes in LUAD and normal tissues. (**B**) Volcano plot exhibiting 25 downregulated and 39 upregulated genes. (**C**) GO enrichment analysis. (**D**) KEGG pathway enrichment analysis.

Sixty-four differentially expressed AAM-related genes were identified in TCGA (Figure 1A), of which 25 were downregulated and 39 were upregulated (Figure 1B). Subsequently, 64 AAM-related genes were used for enrichment analysis. The results of the GO and KEGG analyses revealed that AAM-related genes were focused on various amino acid metabolism pathways (Figure 1C,D). 

### 3.2. Construction of AAMRGS

**Figure 2:**
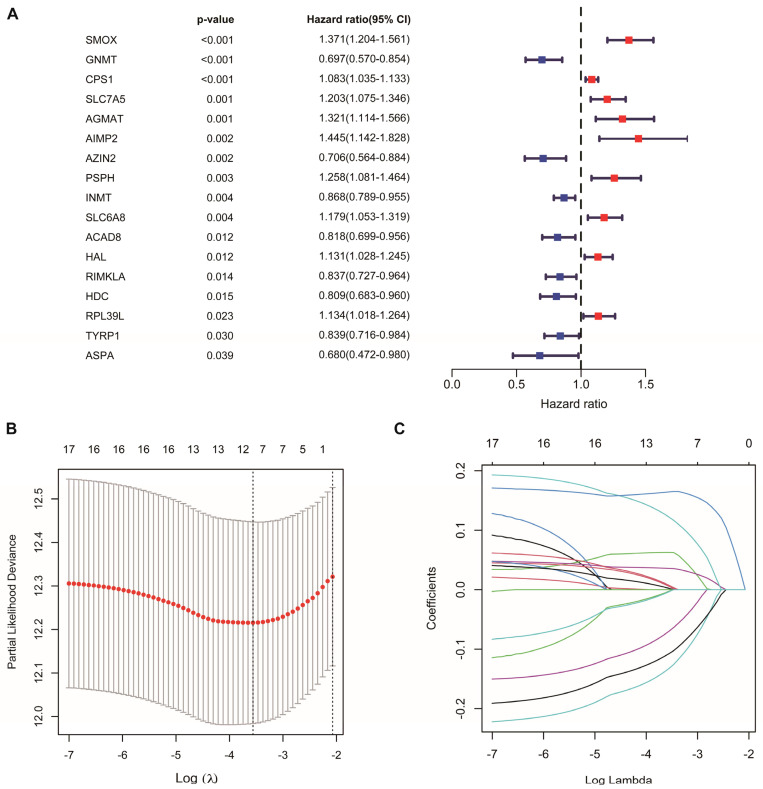
Construction of AAM-related gene signature for LUAD. (**A**) Univariate Cox regression analysis identifying prognostic-related genes. (**B**) Tenfold cross-validation in LASSO model. (**C**) LASSO coefficients of 17 prognostic-related genes.

Univariate Cox regression analysis identified 17 AAM-related genes associated with overall survival (OS) (Figure 2A, Table S2). LASSO Cox regression analysis was used to reduce overfitting effects (Figure 2B,C). Then, the screened genes were carried to the multivariate Cox regression analysis, and six key genes and their coefficients were obtained (Table 1). The risk score for each patient = *CPS1* * 0.057829 − *AZIN2* * 0.208565 − *GNMT* * 0.179454 + *PSPH* * 0.224684 − *RIMKLA* * 0.12884 + *SMOX* * 0.181954.

### 3.3. Prognostic Value of AAMRGS

**Figure 3:**
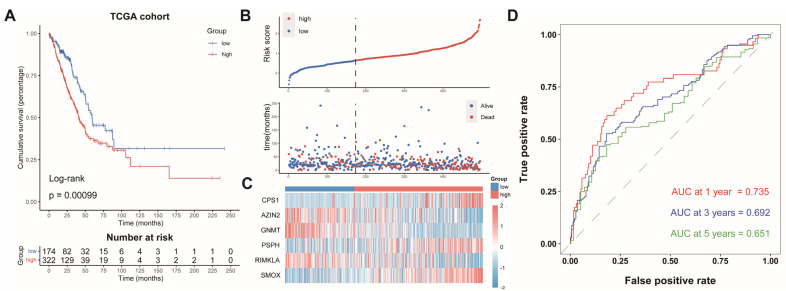
Evaluation and validation of the prognostic value of AAMRGS. (**A**) Kaplan‒Meier survival analysis in the TCGA cohort. (**B**) Distribution of risk scores and OS status in the TCGA cohort. (**C**) Heatmap displaying six AAM-related genes in the TCGA cohort. (**D**) Time-dependent ROC analysis in the TCGA cohort.

The LUAD patients were split into two groups: an AAMRGS-high group (*n* = 322) and an AAMRGS-low group (*n* = 174). The prognosis of the AAMRGS-high group was poorer than that of the AAMRGS-low group (*p* = 0.00099, Figure 3A). Figure 3B displays the risk score and survival status distribution, and significantly more people died in the AAMRGS-high group than in the AAMRGS-low group. The expression of the six key genes in two subgroups was plotted in a heatmap (Figure 3C). The area under curve (AUC) values of the AAMRGS for predicting survival at 1, 3, and 5 years were 0.735, 0.692, and 0.651, respectively (Figure 3D). 

**Figure 4:**
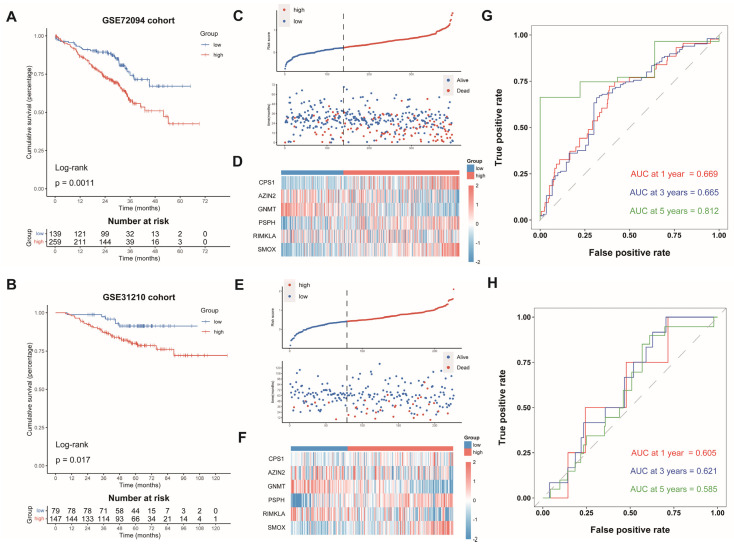
Validation of AAMRGS in GEO datasets. Kaplan‒Meier survival analysis in GSE72094 (**A**) and GSE31210 (**B**). The distribution of risk scores and survival status in GSE72094 (**C**) and GSE31210 (**E**). Heatmap displaying six AAM-related genes in GSE72094 (**D**) and GSE31210 (**F**). Time-dependent ROC analysis in GSE72094 (**G)** and GSE31210 (**H**).

Subsequently, we computed the risk scores of LUAD cases from GSE72094 and GSE31210 on the grounds of the same formula. The patients were split into the AAMRGS-high group and the AAMRGS-low group. The OS was poorer in the AAMRGS-high group compared with that in the AAMRGS-low group. (*p* < 0.05, Figure 4A,B). The risk score and survival status distribution, as well as the heatmap of the expression of the six genes, were similar to those of the TCGA cohort (Figure 4C–F). The AUC values of the signature for predicting survival showed good prediction ability (Figure 4G,H).

### 3.4. Development of a Nomogram Based on AAMRGS

**Figure 5:**
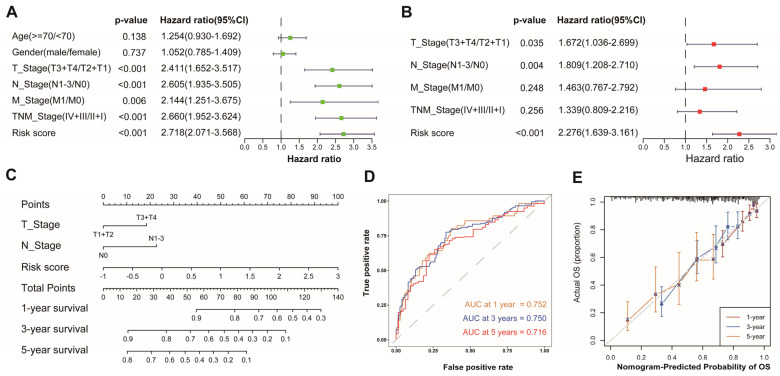
Establishment and assessment of a nomogram in the TCGA cohort. Univariate (**A**) and multivariate (**B**) analyses of risk score and clinicopathological features. (**C**) Nomogram for predicting 1-, 3-, and 5-year survival probability. (**D**) AUC values derived from time-dependent ROC of the nomogram. (**E**) Calibration curves for evaluating the compatibility between the predicted and actual OS.

To evaluate whether the AAMRGS was a reliable prognostic marker for LUAD patients, we performed univariate and multivariate Cox regression analyses successively (Figure 5A,B). The results indicated that the AAMRGS was a dependable prognostic factor for LUAD patients (HR = 2.276, 95% CI: 1.639–3.161, *p* < 0.001). Subsequently, a nomogram was established to predict OS at 1, 3, and 5 years on the basis of the results of the multivariate Cox regression (Figure 5C). The nomogram’s AUC values for predicting 1-, 3-, and 5-year survival were 0.752, 0.750, and 0.716, respectively (Figure 5D). The calibration curves also showed that the predicted and actual OS values at 1, 3, and 5 years were basically consistent (Figure 5E). These outcomes indicated the excellent predictive ability of the nomogram.

### 3.5. Differences in Biological Function and Pathways 

**Figure 6:**
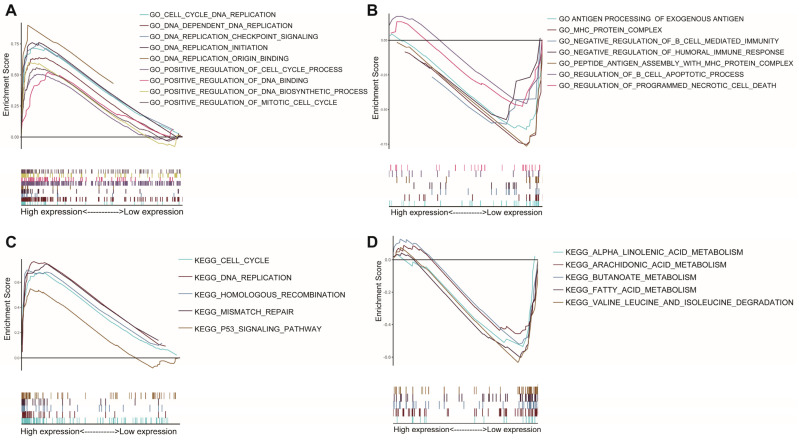
Gene set enrichment analysis between the two AAMRGS subgroups based on the AAMRGS. GO enrichment in the AAMRGS-high group (**A**) and AAMRGS-low group (**B**). KEGG enrichment in the AAMRGS-high group (**C**) and AAMRGS-low group (**D**).

GSEA was used for the two AAMRGS subgroups. According to the GO enrichment analysis, the AAMRGS-high group was mostly enriched in the cell cycle and DNA replication process (Figure 6A). In the AAMRGS-low group, enrichment in B cells mediated immune processes, and antigen presentation was observed (Figure 6B). The KEGG results indicated that the AAMRGS-high group was enriched in the DNA replication, cell cycle, and p53 signaling pathways (Figure 6C), while the AAMRGS-low group showed enrichment mainly in the metabolic processes of a variety of nutrients (Figure 6D).

### 3.6. Analysis of Immune Cell Infiltration and Prediction of Immunotherapy Response

**Figure 7:**
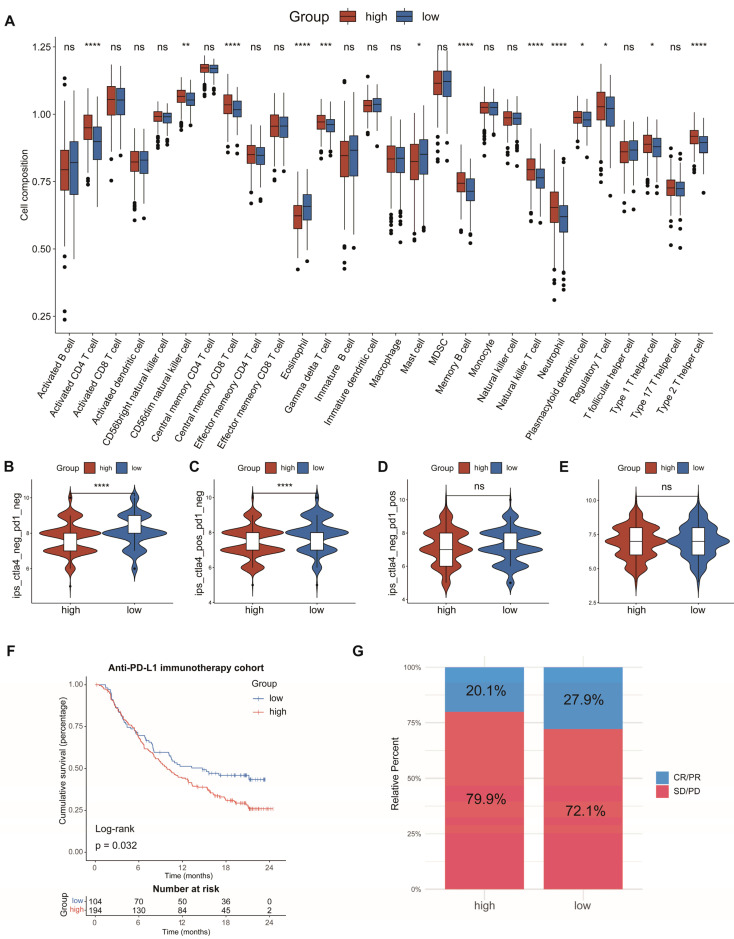
Analysis of immune cell infiltration and prediction of immunotherapy response. (**A**) The differences in the proportions of 28 immune cells between the two subgroups. (**B**) CTLA4_negative_/PD-1_negative_. (**C**) CTLA4_positive_/PD-1_negative_. (**D**) CTLA4_negative_/PD-1_positive_. (**E**) CTLA4_positive_/PD-1_positive_. (**F**) KM survival analysis in the anti-PD-L1 immunotherapy cohort. (**G**) Evaluation of response to anti-PD-L1 immunotherapy. CR, Complete Response; PR, Partial Response; SD, Stable Disease; and PD, Progressive Disease. *P* values were shown as ns: *p >* 0.05; * *p* < 0.05; ** *p* < 0.01; *** *p* < 0.001; **** *p* < 0.0001.

To investigate the connection between the AAMRGS and TME, we analyzed the relationship between the risk score and the level of immune cell infiltration. The analysis of ssGSEA revealed that activated CD4 T cells, gamma delta T cells, natural killer T cells, neutrophils, regulatory T cells, and type 2 T helper cells showed significant infiltration in the AAMRGS-high group. In the AAMRGS-low group, eosinophils and mast cells showed significant infiltration (Figure 7A). The TIMER database is an online analysis website that uses RNA-seq data to analyze the infiltration of immune cells in tumors [24]. Using this database, we examined the association between six key genes and six kinds of immune cells (B cells, CD8+ T cells, CD4+ T cells, macrophages, neutrophils cells, and dendritic cells). The results are shown in Figure S1. To assess the ability of the AAMRGS to forecast the effectiveness of immunotherapy, we analyzed differences in IPS between the two AAMRGS subgroups. The AAMRGS-low group was more sensitive to treatment, with CTLA4_negtive_/PD-1_negtive_ and CTLA4_positive_/PD-1_negtive_ (Figure 7B,C). The sensitivity to immunotherapy was not discernibly different for the CTLA4_negtive_/PD-1_postive_ and CTLA4_postive_/PD-1_postive_ samples between the two subgroups (Figure 7D,E). Subsequently, we utilized the immunotherapy cohort (IMvigor210) to predict patients’ responses to immunotherapy. The prognosis of patients in the AAMRGS-low group was improved (Figure 7F), and more patients responded to immunotherapy (Figure 7G). The above results suggest that the AAMRGS may be a marker of immunotherapy efficacy.

### 3.7. Relationship between AAMRGS and Chemotherapy Drugs

**Figure 8:**
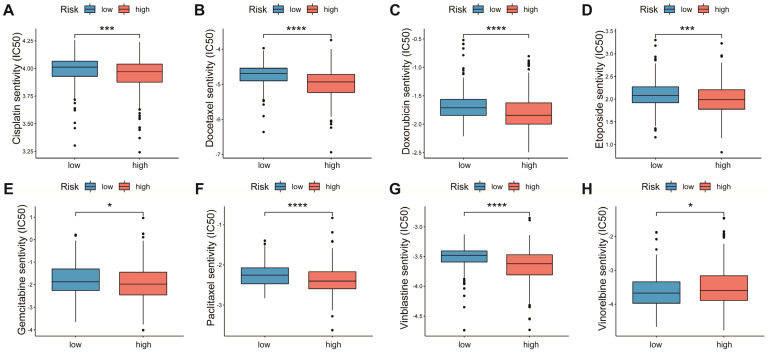
Sensitivity to chemotherapeutic drugs in the two AAMRGS subgroups. (**A**) Cisplatin. (**B**) Docetaxel. (**C**) Doxorubicin. (**D**) Etoposide. (**E**) Gemcitabine. (**F**) Paclitaxel. (**G**) Vinblastine. (**H**) Vinorelbine. *P* values are shown as * *p* < 0.05; *** *p* < 0.001; **** *p* < 0.0001.

Chemotherapy is the main option for patients with advanced LUAD who are not responsive to targeted therapy and immunotherapy. We explored the associations of multiple chemotherapeutic agents with the AAMRGS. Patients in the AAMRGS-high group were more responsive to cisplatin, docetaxel, doxorubicin, etoposide, gemcitabine, paclitaxel, and vinblastine (Figure 8A–G). However, compared with other drugs, the two groups had opposite sensitivities to vinorelbine (Figure 8H). In addition, we also analyzed the sensitivity of all LUAD patients to chemotherapy drugs as a whole (Figure S2). This implied that the AAMRGS-high group of patients would benefit more from chemotherapy. 

## 4. Discussion

Cancers are generally considered to arise from gene mutations in cells that subsequently affect tumor biological behavior, including proliferation, invasion, and metastasis, through alterations at the protein level [25]. In this process, the metabolic reprogramming of amino acids plays a major role [10]. Because malignant tumors have the ability to proliferate indefinitely, they need a large number of amino acids to participate in their own synthesis. Therefore, limiting amino acid intake and targeting key enzymes in amino acid synthesis may become new avenues for cancer therapy. At present, asparaginase has been developed for the treatment of childhood acute lymphoblastic leukemia (ALL) [26]. However, studies targeting amino acid metabolism-related genes in LUAD are rarely mentioned.

In this study, we screened the differentially expressed AAM-related genes in TCGA and constructed an AAMRGS including six genes (*CPS1*, *AZIN2*, *GNMT*, *PSPH*, *RIMKLA*, and *SMOX*) through univariate, LASSO, and multivariate Cox regression analyses. The carbamoyl phosphate synthase encoded by *CPS1* is the first rate-limiting enzyme of the urea cycle. *CPS1* knockdown in LUAD inhibits the JAK/STAT pathway, which is involved in tumor proliferation, differentiation, apoptosis, and immune regulation [27]. Therefore, *CPS1* has the potential to become a new therapeutic target. In non-small cell lung cancer (NSCLC), *AZIN2* overexpression promotes cisplatin resistance [28]. *GNMT* is a tumor suppressor gene in liver cancer, and its high expression can inhibit tumor proliferation [29]. Overexpression of *PSPH* in NSCLC is linked to a bad prognosis [30]. *RIMKLA* is involved in glutamine metabolism, and its role in LUAD is unclear. The AAMRGS showed a good ability to predict prognosis. According to KM survival analysis, a greater risk score was linked to a poorer outcome. Two external GEO datasets verified the accuracy of the signature. Univariate and multivariate Cox regression analyses also suggested that the AAMRGS was a reliable prognostic marker in LUAD. These results provide a reliable basis for the practical application of the AAMRGS. Furthermore, a nomogram was created for easy clinical application.

The GSEA results indicated tumor proliferation and the cell cycle were active in the AAMRGS-high group, which suggests that AAM-related genes play a key role in the metabolic process of tumors, while the AAMRGS-low group was associated with immune-related pathways. Previous studies have shown that amino acid metabolism can reshape the TME by affecting the proliferation and activation of immune cells [31,32,33]. Immune cells in the TME are conducive to strengthening the efficacy of immunotherapy. In this study, ssGSEA showed that eosinophils and mast cells with antitumor immunity function showed significant infiltration in the AAMRGS-low group, while neutrophils, regulatory T cells, and type 2 T helper cells showed significant infiltration in the AAMRGS-high group and played an immunosuppressive role. This confirms that amino acid metabolism can alter the degree of immune cell infiltration, which in turn affects prognosis. The AAMRGS can effectively distinguish different immune states.

In view of the importance of immunotherapy in cancer treatment, subsequently, we analyzed the connection between the AAMRGS and immunotherapy. On the basis of the IPS results, we found that the AAMRGS-low group was more likely to benefit from immunotherapy, largely because the TME was in a state of immune activation. Immunotherapy has become the first-line treatment for various cancers [34]. However, the role of immunotherapy in most patients is limited [35]. It is urgently necessary to develop new biomarkers for immunotherapy efficacy. Our study found that the AAMRGS could predict the response to immunotherapy. The AAMRGS-low group had a better response to anti-PD-L1 immunotherapy. However, some patients with advanced LUAD do not show sensitivity to targeted drugs or immunotherapy. The traditional chemotherapy strategy is the first choice for such patients [36]. In this study, we analyzed the IC50 of traditional chemotherapy drugs, and the results indicated that the patients in the AAMRGS-high group were more responsive to most chemotherapy drugs. The above results may provide a reasonable treatment strategy for LUAD patients. Previous research [37] comprehensively evaluated the expression status of multiple genes through various algorithms, which provided an important basis for precision and individualized treatment. This study provides good evidence for AAMRGS to be used in clinical practice. In the future, we can test samples of LUAD patients for adjuvant treatment to provide more appropriate treatment for patients.

This study has some limitations. First, the validation data only come from public databases, which still need to be validated by multicenter clinical research. Second, the predictive value of the AAMRGS for immunotherapy can only be verified with other types of cancer due to the lack of immunotherapy data for LUAD patients. Additionally, the individual or combined effects of the six genes involved in constituting AAMRGS require further experiments to analyze and verify the results.

## 5. Conclusions

In summary, we analyzed the role of AAM-related genes in LUAD and constructed the AAMRGS to predict prognosis. The AAMRGS could effectively distinguish survival and the state of the TME in LUAD patients. Patients in the AAMRGS-high group were more sensitive to chemotherapy, while those in the AAMRGS-low group were more sensitive to immunotherapy. This provides new insight into the individualized treatment of patients with LUAD.

## Figures and Tables

**Table 1:** Multivariable Cox regression analysis of AAM-related genes in LUAD.

Gene	Coef	*p*-Value	HR	HR.95L	HR.95H
CPS1	0.057829	0.017786	1.059534	1.010056	1.111435
AZIN2	−0.208565	0.079407	0.811748	0.643005	1.024775
GNMT	−0.179454	0.097627	0.835726	0.675848	1.033426
PSPH	0.224684	0.003701	1.251928	1.075694	1.457033
RIMKLA	−0.12884	0.059402	0.879115	0.768904	1.005122
SMOX	0.181954	0.008683	1.199559	1.047138	1.374166

HR: hazard ratio; L: low; H: high.

## Data Availability

The source data and statistical programs for the analysis are available at https://www.jianguoyun.com/p/DRfwZc0QwPGFChj7yd8EIAA (accessed on 24 November 2022).

## Supplementary Materials

The following supporting information can be downloaded at: https://www.mdpi.com/article/10.3390/genes13122295/s1, Figure S1: Relationship between key AAM-related genes and immune cell infiltration from TIMER database. Figure S2: Sensitivity of LUAD samples to eight chemotherapeutic drugs. Table S1: Amino acid metabolism-related genes. Table S2: Univariate Cox regression analysis of differentially expressed AAM-related genes.

## References

[1] Siegel R.L., Miller K.D., Fuchs H.E., Jemal A. (2021). Cancer Statistics, 2021. CA Cancer J. Clin..

[2] Succony L., Rassl D.M., Barker A.P., McCaughan F.M., Rintoul R.C. (2021). Adenocarcinoma spectrum lesions of the lung: Detection, pathology and treatment strategies. Cancer Treat. Rev..

[3] Aberle D.R., Adams A.M., Berg C.D., Black W.C., Clapp J.D., Fagerstrom R.M., Gareen I.F., Gatsonis C., Marcus P.M., The National Lung Screening Trial Research Team (2011). Reduced lung-cancer mortality with low-dose computed tomographic screening. N. Engl. J. Med..

[4] Arbour K.C., Riely G.J. (2019). Systemic Therapy for Locally Advanced and Metastatic Non-Small Cell Lung Cancer: A Review. JAMA.

[5] Hirsch F.R., Scagliotti G.V., Mulshine J.L., Kwon R., Curran W.J., Wu Y.L., Paz-Ares L. (2017). Lung cancer: Current therapies and new targeted treatments. Lancet.

[6] Miller M., Hanna N. (2021). Advances in systemic therapy for non-small cell lung cancer. BMJ.

[7] Kara A., Ozgur A., Nalbantoglu S., Karadag A. (2021). DNA repair pathways and their roles in drug resistance for lung adenocarcinoma. Mol. Biol. Rep..

[8] Balachandran V.P., Gonen M., Smith J.J., DeMatteo R.P. (2015). Nomograms in oncology: More than meets the eye. Lancet Oncol..

[9] Faubert B., Solmonson A., DeBerardinis R.J. (2020). Metabolic reprogramming and cancer progression. Science.

[10] Lieu E.L., Nguyen T., Rhyne S., Kim J. (2020). Amino acids in cancer. Exp. Mol. Med..

[11] Wang W., Zou W. (2020). Amino Acids and Their Transporters in T Cell Immunity and Cancer Therapy. Mol. Cell.

[12] Hensley C.T., Wasti A.T., DeBerardinis R.J. (2013). Glutamine and cancer: Cell biology, physiology, and clinical opportunities. J. Clin. Investig..

[13] Leone R.D., Zhao L., Englert J.M., Sun I.M., Oh M.H., Sun I.H., Arwood M.L., Bettencourt I.A., Patel C.H., Wen J. (2019). Glutamine blockade induces divergent metabolic programs to overcome tumor immune evasion. Science.

[14] Liu J., Shen H., Gu W., Zheng H., Wang Y., Ma G., Du J. (2022). Prediction of prognosis, immunogenicity and efficacy of immunotherapy based on glutamine metabolism in lung adenocarcinoma. Front. Immunol..

[15] Peng H., Wang Y., Luo W. (2020). Multifaceted role of branched-chain amino acid metabolism in cancer. Oncogene.

[16] Ikeda K., Kinoshita M., Kayama H., Nagamori S., Kongpracha P., Umemoto E., Okumura R., Kurakawa T., Murakami M., Mikami N. (2017). Slc3a2 Mediates Branched-Chain Amino-Acid-Dependent Maintenance of Regulatory T Cells. Cell Rep..

[17] Subramanian A., Tamayo P., Mootha V.K., Mukherjee S., Ebert B.L., Gillette M.A., Paulovich A., Pomeroy S.L., Golub T.R., Lander E.S. (2005). Gene set enrichment analysis: A knowledge-based approach for interpreting genome-wide expression profiles. Proc. Natl. Acad. Sci. USA.

[18] Ritchie M.E., Phipson B., Wu D., Hu Y., Law C.W., Shi W., Smyth G.K. (2015). limma powers differential expression analyses for RNA-sequencing and microarray studies. Nucleic Acids Res..

[19] Yu G., Wang L.G., Han Y., He Q.Y. (2012). clusterProfiler: An R package for comparing biological themes among gene clusters. Omics A J. Integr. Biol..

[20] Hanzelmann S., Castelo R., Guinney J. (2013). GSVA: Gene set variation analysis for microarray and RNA-seq data. BMC Bioinform..

[21] Charoentong P., Finotello F., Angelova M., Mayer C., Efremova M., Rieder D., Hackl H., Trajanoski Z. (2017). Pan-cancer Immunogenomic Analyses Reveal Genotype-Immunophenotype Relationships and Predictors of Response to Checkpoint Blockade. Cell Rep..

[22] Mariathasan S., Turley S.J., Nickles D., Castiglioni A., Yuen K., Wang Y., Kadel E.E., Koeppen H., Astarita J.L., Cubas R. (2018). TGFbeta attenuates tumour response to PD-L1 blockade by contributing to exclusion of T cells. Nature.

[23] Geeleher P., Cox N., Huang R.S. (2014). pRRophetic: An R package for prediction of clinical chemotherapeutic response from tumor gene expression levels. PLoS ONE.

[24] Li T., Fan J., Wang B., Traugh N., Chen Q., Liu J.S., Li B., Liu X.S. (2017). TIMER: A Web Server for Comprehensive Analysis of Tumor-Infiltrating Immune Cells. Cancer Res..

[25] Bignell G.R., Greenman C.D., Davies H., Butler A.P., Edkins S., Andrews J.M., Buck G., Chen L., Beare D., Latimer C. (2010). Signatures of mutation and selection in the cancer genome. Nature.

[26] Avramis V.I., Panosyan E.H. (2005). Pharmacokinetic/pharmacodynamic relationships of asparaginase formulations: The past, the present and recommendations for the future. Clin. Pharmacokinet..

[27] Celiktas M., Tanaka I., Tripathi S.C., Fahrmann J.F., Aguilar-Bonavides C., Villalobos P., Delgado O., Dhillon D., Dennison J.B., Ostrin E.J. (2017). Role of CPS1 in cell growth, metabolism and prognosis in LKB1-Inactivated Lung adenocarcinoma. J. Natl. Cancer Inst..

[28] Shi Q., Chen Q., Zhou Z., Zheng X., Huang X., Fang M., Hu Y., Song L., Yang H., Chen Q. (2021). Hypoxia-induced antizyme inhibitors 2 regulates cisplatin resistance through epithelia-mesenchymal transition pathway in non-small cell lung cancer. Pulm. Pharmacol. Ther..

[29] Yen C.H., Lu Y.C., Li C.H., Lee C.M., Chen C.Y., Cheng M.Y., Huang S.F., Chen K.F., Cheng A.L., Liao L.Y. (2012). Functional characterization of glycine N-methyltransferase and its interactive protein DEPDC6/DEPTOR in hepatocellular carcinoma. Mol. Med..

[30] Liao L., Ge M., Zhan Q., Huang R., Ji X., Liang X., Zhou X. (2019). PSPH Mediates the Metastasis and Proliferation of Non-small Cell Lung Cancer through MAPK Signaling Pathways. Int. J. Biol. Sci..

[31] Bian Y., Li W., Kremer D.M., Sajjakulnukit P., Li S., Crespo J., Nwosu Z.C., Zhang L., Czerwonka A., Pawlowska A. (2020). Cancer SLC43A2 alters T cell methionine metabolism and histone methylation. Nature.

[32] Carr E.L., Kelman A., Wu G.S., Gopaul R., Senkevitch E., Aghvanyan A., Turay A.M., Frauwirth K.A. (2010). Glutamine uptake and metabolism are coordinately regulated by ERK/MAPK during T lymphocyte activation. J. Immunol..

[33] Geiger R., Rieckmann J.C., Wolf T., Basso C., Feng Y., Fuhrer T., Kogadeeva M., Picotti P., Meissner F., Mann M. (2016). L-Arginine Modulates T Cell Metabolism and Enhances Survival and Anti-tumor Activity. Cell.

[34] Yi M., Zheng X., Niu M., Zhu S., Ge H., Wu K. (2022). Combination strategies with PD-1/PD-L1 blockade: Current advances and future directions. Mol. Cancer.

[35] Herbst R.S., Morgensztern D., Boshoff C. (2018). The biology and management of non-small cell lung cancer. Nature.

[36] Pirker R. (2020). Chemotherapy remains a cornerstone in the treatment of nonsmall cell lung cancer. Curr. Opin. Oncol..

[37] Liu S.Y., Bao H., Wang Q., Mao W.M., Chen Y., Tong X., Xu S.T., Wu L., Wei Y.C., Liu Y.Y. (2021). Genomic signatures define three subtypes of EGFR-mutant stage II-III non-small-cell lung cancer with distinct adjuvant therapy outcomes. Nat. Commun..

