# Supplementary figures and images for: Identification of an Amino Acid Metabolism-Related Gene Signature for Predicting Prognosis in Lung Adenocarcinoma
Source: Genes (Basel). 2022 Dec 6;13(12):2295. doi: 10.3390/genes13122295 (PMC9778477; doi:10.3390/genes13122295)

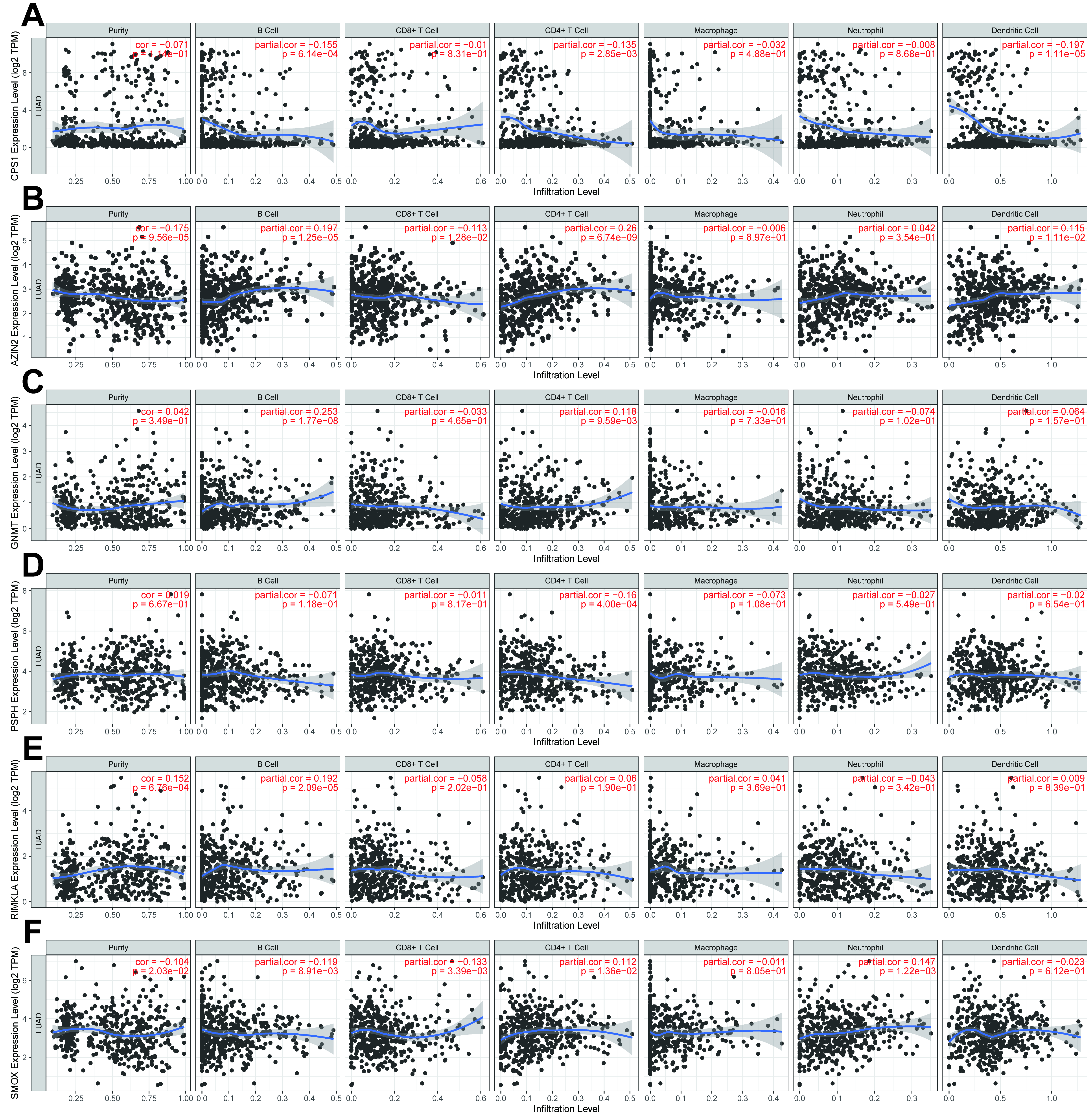

Supplement: Supplementary file 1 [file genes-13-02295-s001.zip › FigureS1.tif]

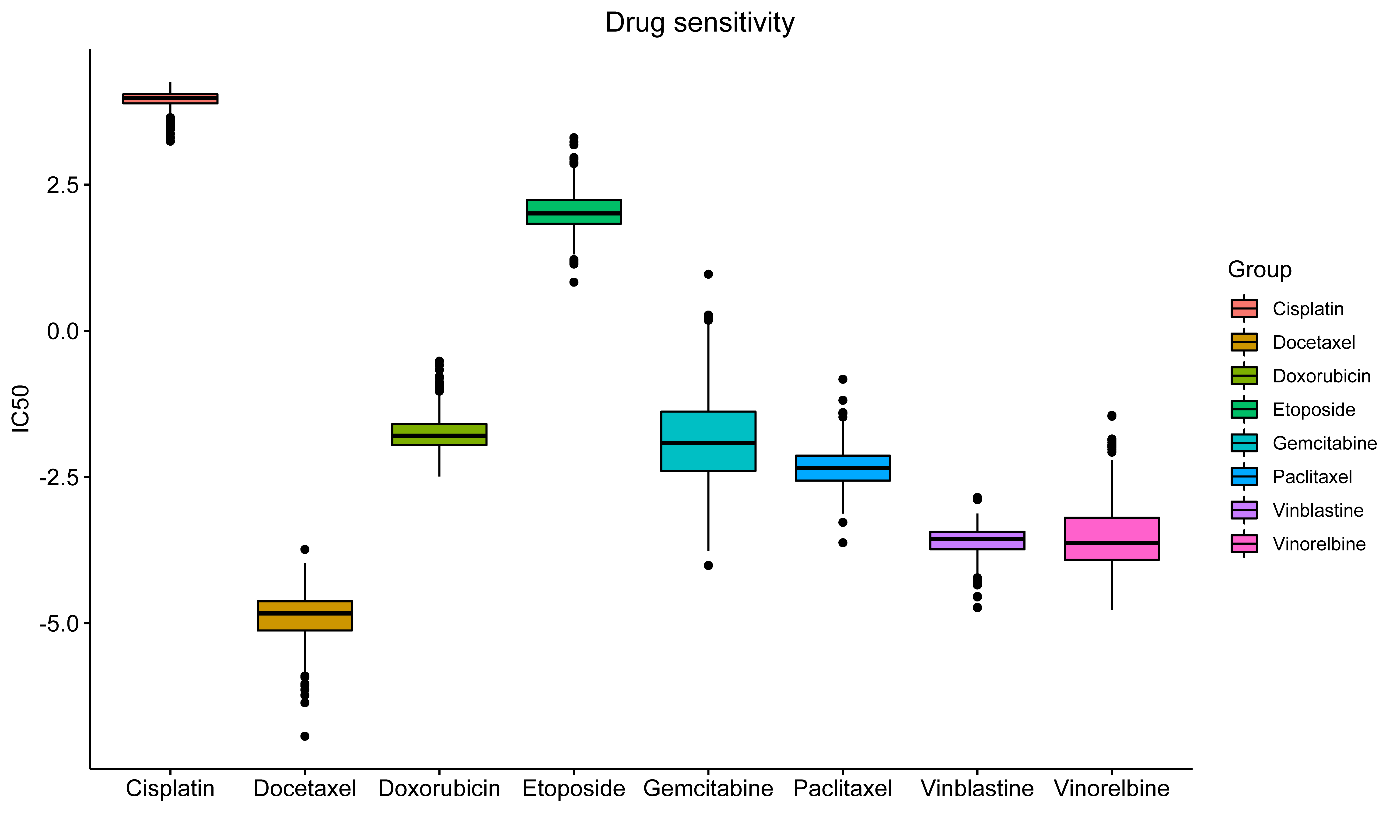

Supplement: Supplementary file 1 [file genes-13-02295-s001.zip › FigureS2.tif]
